# Correction of vaccine effectiveness derived from test-negative case–control studies

**DOI:** 10.1186/s12874-023-01962-0

**Published:** 2023-06-10

**Authors:** Farrokh Habibzadeh

**Affiliations:** Global Virus Network, Middle East Region, Shiraz, Iran

**Keywords:** Vaccines, Case–control studies, Diagnostic tests, Sensitivity and specificity, SARS-CoV-2

## Abstract

**Background:**

Determining the vaccine effectiveness (*VE*) is an important part of studying every new vaccine. Test-negative case–control (TNCC) studies have recently been used to determine the *VE*. However, the estimated *VE* derived from a TNCC design depends on the test sensitivity and specificity. Herein, a method for correction of the value of *VE* derived from a TNCC study is presented.

**Methods:**

An analytical method is presented to compute the corrected *VE* based on the sensitivity and specificity of the diagnostic test utilized. To show the application of the method proposed, a hypothetical TNCC study is presented. In this in silico study, 100 000 individuals referring to a healthcare system for COVID-19-like illness were tested with diagnostic tests with sensitivities of 0.6, 0.8, and 1.0, and specificities ranging from 0.85 to 1.00. A vaccination coverage of 60%, an attack rate of 0.05 for COVID-19 in unvaccinated group, and a true *VE* of 0.70, were assumed. In this simulation, a COVID-19-like illness with an attack rate of 0.30 could also affect all the studied population regardless of their vaccination status.

**Results:**

The observed *VE* ranged from 0.11 (computed for a test sensitivity of 0.60 and specificity of 0.85) to 0.71 (computed for a test sensitivity and specificity of 1.0). The mean computed corrected *VE* derived from the proposed method was 0.71 (the standard deviation of 0.02).

**Conclusions:**

The observed *VE* derived from TNCC studies can be corrected easily. An acceptable estimate for *VE* can be computed regardless of the diagnostic test sensitivity and specificity used in the study.

**Supplementary Information:**

The online version contains supplementary material available at 10.1186/s12874-023-01962-0.

## Background

Vaccines have had a significant impact on global health. Some vaccines provide lifelong protection against infections; others confer temporary protection. Many circulating pathogens change over time, which affects the effectiveness of vaccines made against them [1, 2], which is why the effectiveness of most vaccines (*e.g.*, influenza vaccine) needs to be updated regularly [3]. Determining and regular monitoring of vaccine effectiveness (*VE*) is thus an integral part of studying every vaccine.

*VE* is a measure reflecting how well a vaccine prevents illness, hospitalization, or death in those who were vaccinated compared to unvaccinated individuals [4]. It is commonly expressed as a percentage reduction in the risk (incidence) of the disease in vaccinated *vs*. unvaccinated people. For example, if the incidence of a certain disease is 0.05 in unvaccinated people and vaccination decreases it to 0.015, then the vaccination decreases the risk by 70% (*i.e.*, 0.70), hence, the corresponding *VE* is 0.70. Mathematically, *VE* can be calculated as follows [5, 6]:

1 $$\begin{aligned} VE &= \frac{{AR_{unvac} - AR_{vac} }}{{AR_{unvac} }} \\ &= 1 - \frac{{AR_{vac} }}{{AR_{unvac} }} \\ &= 1 - RR \\ \end{aligned}$$

where *AR*_*unvac*_ and *AR*_*vac*_ are the attack rates of the infection in the unvaccinated and vaccinated individuals, and *RR* is the relative risk. Observational studies are commonly used to determine the *VE* [7]. A cohort study is the only study design that can accurately provide the *AR* of the disease of interest in the vaccinated and unvaccinated groups and thus, is the best type of observational studies for calculation of the *VE*. However, given the low *AR* for many infectious disease, the odds ratio (*OR*) derived from a case–control study can be considered an acceptable estimate for the *RR* [8]. Therefore, a case–control study can also be used for determination of the *VE* [7].

Over the recent years, test-negative case–control (TNCC) studies have commonly been used to determine the *VE* [3]. Technically, a TNCC study has a case–control design except that the way the cases and controls are recruited is different. For example, to determine the *VE* of a new vaccine developed against SARS-CoV-2 using a TNCC design, cases and controls are selected from a cohort of patients attending a healthcare center because of a COVID-19-like illness; those who are tested positive for SARS-CoV-2 are considered “cases;” the remaining with a negative test, “controls” (Table 1) [5, 9]. The *VE* is then [7]:

2 $$\begin{aligned} VE &= 1 - RR \\ &\approx 1 - OR \\ &\approx 1 - \frac{{{\raise0.7ex\hbox{$a$} \!\mathord{\left/ {\vphantom {a b}}\right.\kern-0pt} \!\lower0.7ex\hbox{$b$}}}}{{{\raise0.7ex\hbox{$c$} \!\mathord{\left/ {\vphantom {c d}}\right.\kern-0pt} \!\lower0.7ex\hbox{$d$}}}} \\ \end{aligned}$$

**Table 1:** The general form of a test-negative case–control study in the whole study population — patients who sought and those who did not seek medical care stratified by vaccination status. Note that we do not have any information about those who did not seek medical care (last two columns)

Vaccinated	Seeking Medical Care		Total	Not Seeking Medical Care	
	**Test + *ve***^a^	**Test *–ve***^b^		**Test + *ve***	**Test *–ve***
Yes	*a*	*b*	*a* + *b*	*e*	*f*
No	*c*	*d*	*c* + *d*	*g*	*h*
**Total**	*a* + *c*	*b* + *d*	*n*	*e* + *g*	*f* + *h*

^a^ + *ve*: Positive

^b^ *–ve*: Negative

To have a valid *VE*, a valid *OR* is needed, which in turn necessitates equality of the *OR*s computed for those who seek medical care and for those who do not seek medical care [10], that is (Table 1):

3 $$\frac{{{\raise0.7ex\hbox{$a$} \!\mathord{\left/ {\vphantom {a b}}\right.\kern-0pt} \!\lower0.7ex\hbox{$b$}}}}{{{\raise0.7ex\hbox{$c$} \!\mathord{\left/ {\vphantom {c d}}\right.\kern-0pt} \!\lower0.7ex\hbox{$d$}}}} = \frac{{{\raise0.7ex\hbox{$e$} \!\mathord{\left/ {\vphantom {e f}}\right.\kern-0pt} \!\lower0.7ex\hbox{$f$}}}}{{{\raise0.7ex\hbox{$g$} \!\mathord{\left/ {\vphantom {g h}}\right.\kern-0pt} \!\lower0.7ex\hbox{$h$}}}}$$

TNCC design is relatively cheaper and faster to conduct than cohort and traditional case–control studies [9]. Nonetheless, the estimated *VE* value derived from a TNCC design depends on the test sensitivity (*Se*), the probability that a diseased person becomes test-positive, and more seriously on the test specificity (*Sp*), the probability that a disease-free person becomes test-negative [7, 11, 12]. But, the *Se* and *Sp* of most diagnostic tests are not 1.0 (*i.e.*, 100%); there are almost always false-positive and false-negative results that cause misclassification problem [11, 13]. Herein, it is meant to present a method for computation of the *VE* based on the results of a TNCC design regardless of the *Se* and *Sp* of the diagnostic test used in the study.

## Methods

### The proposed correction method

Suppose that a TNCC study was conducted to determine the effectiveness of a new vaccine against SARS-CoV-2 and tested a group of patients attended a healthcare center with COVID-19-like illness. Also, suppose that the results in their parametric form are presented in Table 1. Let us focus on a single row of Table 1, for instance, those vaccinated. If all those who attended the healthcare center were considered a cohort of people with COVID-19-like illness, then the apparent prevalence of COVID-19 in the vaccinated patients who sought medical care is [14]:

4 $$pr = \frac{a}{a + b}$$

Note that *pr* is a true estimation of COVID-19 prevalence neither in the whole population nor in patients who sought medical care, as the diagnostic test used for the diagnosis of COVID-19 was presumably not perfect; there were false-positive and false-negative results. The true prevalence (*π*), the prevalence had a perfect diagnostic test with a *Se* and *Sp* of 1.0 (*i.e.*, 100%) been used, is [14]:

5 $$\begin{aligned} \pi &= \frac{pr + Sp - 1}{{Se + Sp - 1}} \\ &= \frac{{\frac{o}{1 + o} + Sp - 1}}{Se + Sp - 1} \\ \end{aligned}$$

where *o* represents odds corresponding to *pr*, *a*/*b*. The true odds (*ω*) of COVID-19 in the vaccinated patients who sought medical care is then:

6 $$\begin{aligned} \omega &= \frac{\pi }{1 - \pi } \\ &= \frac{{Sp\,\left( {1 + o} \right) - 1}}{{Se\,\left( {1 + o} \right) - o}} \\ \end{aligned}$$

In the same way, the odds in the unvaccinated patients who sought medical care (*c*/*d*), can be derived. From Eq. 2, the observed *VE* is:

7 $$\begin{aligned} VE_{obs} &= 1 - OR_{obs} \\ &= 1 - \frac{{o_{vac} }}{{o_{unvac} }} \\ \end{aligned}$$

Using Eq. 6, the corrected *VE* is then:

8 $$\begin{aligned} VE_{cor} &= 1 - OR_{cor} \\ &= 1 - \frac{{\omega_{vac} }}{{\omega_{unvac} }} \\ &= 1 - \left[ {{{\frac{{Sp\,\left( {1 + o_{vac} } \right) - 1}}{{Se\,\left( {1 + o_{vac} } \right) - o_{vac} }}} \mathord{\left/ {\vphantom {{\frac{{Sp\,\left( {1 + o_{vac} } \right) - 1}}{{Se\,\left( {1 + o_{vac} } \right) - o_{vac} }}} {\frac{{Sp\,\left( {1 + o_{unvac} } \right) - 1}}{{Se\,\left( {1 + o_{unvac} } \right) - o_{unvac} }}}}} \right. \kern-0pt} {\frac{{Sp\,\left( {1 + o_{unvac} } \right) - 1}}{{Se\,\left( {1 + o_{unvac} } \right) - o_{unvac} }}}}} \right] \\ &= \frac{{\left( {o_{unvac} - o_{vac} } \right)\left( {Se + Sp - 1} \right)}}{{\left( {o_{unvac} Sp + Sp - 1} \right)\left[ {o_{vac} \left( {Se - 1} \right) + Se} \right]}} \\ \end{aligned}$$

where the subscripts “*vac*” and “*unvac*” represent the variable in the vaccinated and unvaccinated patients who sought medical care for the COVID-19-like illness, respectively. Variance of the corrected *VE* can be computed too (see Supplementary Materials).

### A hypothetical in silico case study

Let us examine the results of the application of the above scenario in an in silico study. Suppose that we want to conduct a TNCC study on a sample of 100 000 individuals who sought medical care for a COVID-19-like illness. Let 60% of the study population had been vaccinated (vaccine coverage) and that the true *VE* be 0.70. Assume diagnostic tests with different combinations of *Se* (0.6, 0.8, and 1.0) and *Sp* values (ranging from 0.85 to 1.00) for the diagnosis of SARS-CoV-2 were used. Furthermore, suppose that the test *Se* and *Sp* were not different in vaccinated and unvaccinated groups, that COVID-19-like illness affects people living in the study community independent of whether they have already been infected with SARS-CoV-2 or not; that the SARS-CoV-2 has an *AR* of 5% in unvaccinated individuals [15]; and that the *AR* of the COVID-19-like illness is 30% (consistent with the *AR* of non-influenza flu-like illness seen during a cold season) [16]. Moreover, to make things simple, assume that the *AR* of the COVID-19-like illness does not depend on the vaccination status of studied people, duration since vaccination, age, and other variables. Severity of the COVID-19 outcomes (*e.g.*, needing hospitalization or admission in intensive care units) was not taken into account in the current in silico study, as it evidently does not significantly affect the healthcare-seeking behavior of people so that Eq. 3 holds, regardless of the disease severity [17]. A piece of code developed in *R* (*R* software version 4.1.0, *R* Project for Statistical Computing) was used for the simulation (see Supplementary Materials).

## Results and Discussion

Figure 1 shows the apparent and corrected *VE* values for various combinations of test *Se* and *Sp* values used in the in silico TNCC study. The observed *VE* ranged from 0.11 (computed for a test *Se* of 0.60 and *Sp* of 0.85) to 0.71 (computed for a test *Se* and *Sp* of 1.0, a perfect test). The mean computed corrected *VE* derived from the proposed method (Eq. 8) was 0.71 (the standard deviation of 0.02); the corrected value ranged from 0.67 to 0.76. The variation observed was probably attributed to the sampling error in the simulation.

**Fig. 1:**
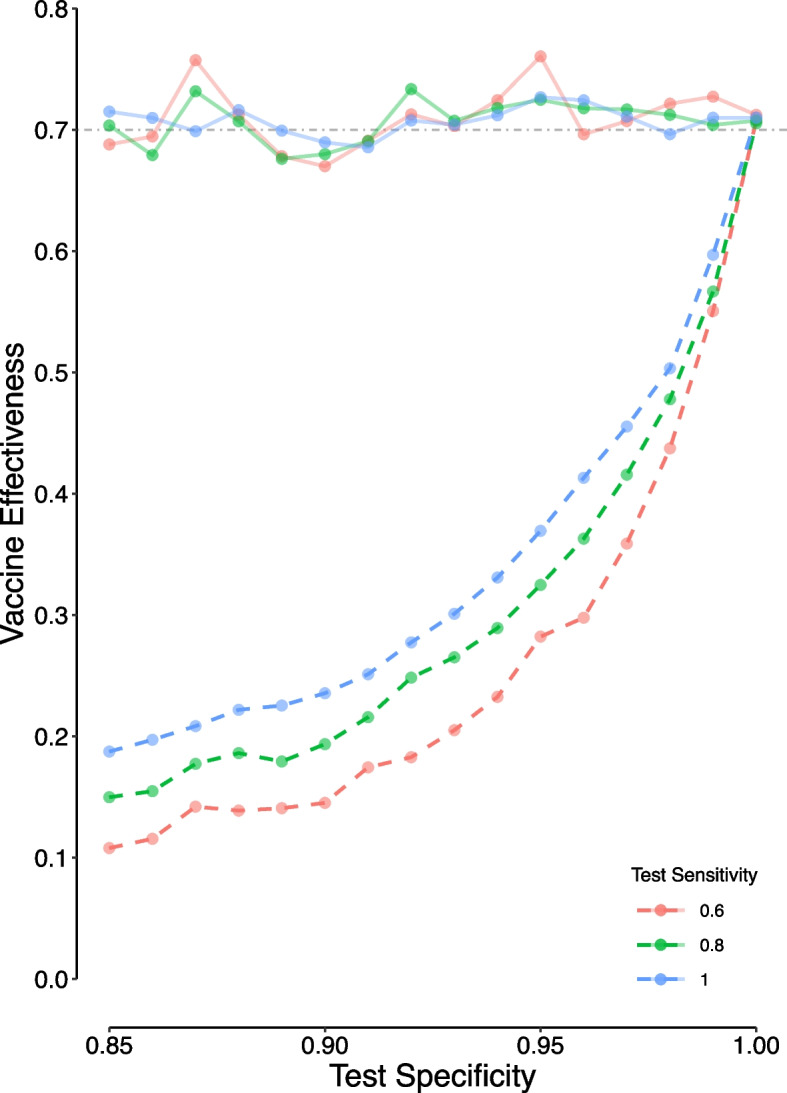
The apparent (dashed lines) and corrected (solid lines) vaccine effectiveness derived from in silico test-negative case–control studies using diagnostic tests with different sensitivities and specificities. In each study 100 000 individuals were examined assuming an attack rate of 5% for SARS-CoV-2 infection in unvaccinated individuals, an attack rate of 30% for the COVID-19-like illness, and a vaccination coverage of 60%. The horizontal dash-dotted gray line represents the true vaccine effectiveness of 0.70

The gross difference between the apparent and corrected *VE* was due to misclassification of patients [13]. The false-positive rate could be decreased by increasing the test *Sp* by changing the cut-off value for tests with continuous results [11]. Should a perfect test (*Se* and *Sp* of 1.0) have been used instead, no false results occurred at all and the apparent *VE* was equal to the true *VE* (Fig. 1). The corrected values computed were just a little bit different from the true *VE* of 0.70. As it has been shown earlier [7], the test *Sp* is much more important than the test *Se*; if a test with a very high *Sp* is used, the observed *VE* is a good estimate of the correct value regardless of the test *Se*, whereas it is not correct for a test with even a *Se* of 1.0 (Fig. 1).

Table 2A shows one example of the data generated in the simulation. Using Eq. 2, the apparent *VE* is 0.18. Plugging in the values in Eq. 8 gives a corrected *VE* of 0.69. The gross difference between the apparent and corrected *VE* is due to the presence of a large number of people with false-positive results (for using a test with lower *Sp*); 5683 in vaccinated and 3206 in unvaccinated groups (Table 2A). The false-positive rate could be decreased by increasing the test *Sp* by changing the cut-off value for tests with continuous results [11]. Should a perfect test have been used instead, no false results occurred at all and the apparent *VE* was equal to the true *VE* (Table 2B). Using Eq. 8, the corrected *VE*, the value if a perfect test would have been used, can be calculated.

**Table 2:** Results of the case study for two conditions — A) when a test with a sensitivity of 0.70 and specificity of 0.90 is used, and B) when a perfect test (sensitivity and specificity of 1.0) is used. The underlined numbers are false-positive results

**A**	**Test**		**Total**
**Vaccinated**	**Positive**	**Negative**	
Yes	910 + 5683	53 342	59 935
No	2039 + 3206	34 820	40 065
**Total**			100 000
**B**	**Test (Disease)**		**Total**
**Vaccinated**	**Positive**	**Negative**	
Yes	910	59 025	59 935
No	2039	38 026	40 065
**Total**	2949	97 051	100 000

The corrected value of 0.71 in this case, as well as the mean corrected *VE* of 0.71 derived from the simulation, is a little bit higher than the true *VE* of 0.70. This is because of using *OR* derived from TNCC design, which is only an estimation of *RR* (Eq. 2). This is in fact true for any case–control studies [8]. However, as long as the *AR* of the disease of interest is small, *OR* is an acceptable estimate for *RR*.

The vaccine coverage in the population was 0.60 (Table 2). The disease *AR* was 3% in the whole study population (Table 2B). This estimation was correct, because the *AR* is different in unvaccinated and vaccinated group. Given the coverage of vaccination, the true *VE*, and the *AR* in unvaccinated and vaccinated groups, the *AR* in the whole population is:

9 $$\begin{aligned} AR_{obs} &= AR_{unvac} \,\frac{{n_{unvac} }}{n} + AR_{vac} \,\frac{{n_{vac} }}{n} \\ &= AR_{unvac} \,\left( {1 - Coverage} \right) + AR_{unvac} \,\left( {1 - VE} \right)Coverage \\ \end{aligned}$$

where *n* is the population size, and *Coverage*, the vaccination coverage. Plugging in the values (*AR*_*unvac*_ of 0.05, *Coverage* of 0.60, and *VE* of 0.70) gives an observed *AR* of 3%.

This study had some limitations. Assuming that the test *Se* and *Sp* were not different in vaccinated and unvaccinated groups, that COVID-19-like illness affects people living in the study community independent of whether they have already been infected with SARS-CoV-2 or not, that vaccination does not affect the disease severity in COVID-19 breakthrough infections [18], and that the *AR* of the COVID-19-like illness does not depend on the vaccination status of studied people, duration since vaccination, age, and other variables, might be oversimplification of the situation. More complex simulations should be designed to assess the possible effects of these factors.

Although TNCC design may diminish the effect of many confounding variables and selection bias attributable to differential recall of the exposure compared with traditional case–control design, it cannot completely eliminate the effects of all confounders [9, 19]. TNCC has the advantage over cohort and traditional case–control studies in that it requires fewer resources and can be conducted within a short period [7]. TNCC and traditional case–control study basically share the same design and thus expectedly have similar biases — for instance, both designs provide *OR*, as an estimate for *RR*, a presumption that is only true with low *AR* values [8].

## Conclusions

It was shown that the correct value of *VE* can be computed regardless of the *Se* and *Sp* of the diagnostic test to be used in a TNCC study. The computed value and its precision depend on the odds of a positive test in vaccinated and unvaccinated patients who sought medical care and the test *Se* and *Sp* (Eq. 8) as well as their variance (see Supplementary Materials). Therefore, although the *Se* and *Sp* of the test utilized might not be important, their precisions are. The *Se* and *Sp* of the test to be used in TNCC studies are better to be estimated in large validity studies. So far, researchers had to utilize highly specific (and most often, sensitive) tests in TNCC designs to come up with an acceptable estimate for the *VE*. Employing the proposed method, it is just enough to use tests with known *Se* and *Sp* values, no matter how much they are. TNCC is not only used to determine *VE*, but also has other applications including risk assessment in other settings such as antibiotic resistance [20], and venous thrombosis [21], to name only a few. The proposed correction method may be applied to the results of these studies too.

## Supplementary Information

**Additional file 1.**

## Data Availability

All data generated or analyzed during this study as well as the *R* codes are included in this published article and its supplementary information files.

## Abbreviations

*AR*: Attack rate
*RR*: Relative risk
*OR*: Odds ratio
*VE*: Vaccine effectiveness
TNCC: Test-negative case–control
SARS-CoV-2: Severe acute respiratory syndrome coronavirus 2
COVID-19: Coronavirus disease 2019
*Se*: Sensitivity
*Sp*: Specificity
*pr*: Prevalence
*π*: True prevalence
