# Supplementary material for: Correction of vaccine effectiveness derived from test-negative case–control studies
Source: BMC Med Res Methodol. 2023 Jun 10;23:137. doi: 10.1186/s12874-023-01962-0 (PMC10257167; doi:10.1186/s12874-023-01962-0)
Supplement: Supplementary file 1 — Additional file 1. [file 12874_2023_1962_MOESM1_ESM.docx]

**SUPPLEMENTARY MATERIALS**

**Supplementary Methods***Variance of the Corrected Vaccine Effectiveness*

It was shown that the corrected vaccine effectiveness (*VE*) can be derived using the following equation (Eq 8 in the article):

$$VE_{cor}=1-\left[\frac{Sp \left(1+o_{vac}\right)-1}{Se \left(1+o_{vac}\right)-o_{vac}}/\frac{Sp \left(1+o_{unvac}\right)-1}{Se \left(1+o_{unvac}\right)-o_{unvac}}\right]$$

where *VE_cor_* is the corrected *VE*; *Se*, test sensitivity; *Sp*, test specificity; and *o*, odds of the disease in vaccinated and unvaccinated groups (designated by its subscript). Assuming that *Se*, *Sp*, and odds are independent, using a first-order Taylor series expansion [1], the variance in the corrected VE is then:

$$\begin{matrix} SE_{VE_{cor}}^{2}=\left(\frac{\partial VE_{cor}}{\partial Se}\right)^{2}SE_{Se}^{2}+\left(\frac{\partial VE_{cor}}{\partial Sp}\right)^{2}SE_{Sp}^{2}+\left(\frac{\partial VE_{cor}}{\partial o_{vac}}\right)^{2}SE_{o_{vac}}^{2}+\left(\frac{\partial VE_{cor}}{\partial o_{unvac}}\right)^{2}SE_{o_{unvac}}^{2} \\ =\left(\frac{\left(o_{vac}-o_{unvac}\right)\left[Sp\left(o_{vac}+1\right)-1\right]}{\left[Se\left(o_{vac}+1\right)-o_{vac}\right]^{2} \left[Sp\left(o_{unvac}+1\right)-1\right]}\right)^{2}SE_{Se}^{2}+ \\ \left(\frac{\left(o_{vac}-o_{unvac}\right)\left[Se\left(o_{unvac}+1\right)-o_{unvac}\right]}{\left[Se\left(o_{vac}+1\right)-o_{vac}\right] \left[Sp\left(o_{unvac}+1\right)-1\right]^{2}}\right)^{2}SE_{Sp}^{2}+ \\ \left(\frac{\left(Se+Sp-1\right)\left[Se\left(o_{unvac}+1\right)-o_{unvac}\right]}{\left[1-Sp\left(o_{unvac}+1\right)\right] \left[Se\left(o_{vac}+1\right)-o_{vac}\right]^{2}}\right)^{2}SE_{o_{vac}}^{2}+ \\ \left(\frac{\left(Se+Sp-1\right)\left[Sp\left(o_{Vac}+1\right)-1\right]}{\left[Se\left(o_{vac}+1\right)-o_{vac}\right] \left[Sp\left(o_{unvac}+1\right)-1\right]^{2}}\right)^{2}SE_{o_{Unvac}}^{2} \end{matrix}$$

**References**

1. Champac V, Gervacio JG: **Appendix A: Variance of a Function of Random Variables Approximated with Taylor’s Theorem**. In: *Timing Performance of Nanometer Digital Circuits Under Process Variations.* edn. Edited by Champac V, Gervacio JG: Springer; 2018.

***R* codes for the simulation**

library("ggplot2")

library("ggh4x")

#------------------------------------------------------------------------------

N <- 400000 # Population size

v_coverage <- 0.6 # Vaccine coverage

n <- 100000 # Sample size (in each group)

AR <- 0.05 # Attack Rate in unvaccinated

AR_ill <- 0.30 # Attack Rate of flu-like illness

VE <- 0.70 # Vaccine effectiveness

set.seed(123)

dat <- data.frame(dis = rep(0, N), Vac = rep(0, N), ill = rep(0, N))

dat$Vac[1:round(v_coverage*N)] <- 1

dat <- dat[sample(nrow(dat)),] # Shuffle dat

dat$ill[1:round(AR_ill*N)] <- 1

dat <- dat[sample(nrow(dat)),] # Shuffle dat

len <- length(dat[dat$Vac == 0,]$dis)

dat[dat$Vac == 0,]$dis[1:round(AR*len)] <- 1

len <- length(dat[dat$Vac == 1,]$dis)

dat[dat$Vac == 1,]$dis[1:round(AR*(1-VE)*len)] <- 1

dat$Vac = factor(dat$Vac, labels=c("Unvaccinated", "Vaccinated"))

dat$dis = factor(dat$dis, labels=c("Undiseased", "Diseased"))

dat$ill = factor(dat$ill, labels=c("Not ill", "Ill"))

dat <- dat[sample(nrow(dat)),] # Shuffle dat

dat_tmp <- dat

df <- data.frame(se=numeric(), sp=numeric(), ve=numeric(), vec=numeric())

for(se in seq(0.6, 1, by = 0.2)){

dat <- dat_tmp

for(sp in seq(0.85, 1, by = 0.01)){

dat_dis <- dat[dat$dis == "Diseased",]

len <- nrow(dat_dis)

dat_dis$test <- 0

dat_dis$test[1:round(se*len)] <- 1 # True +ve

dat_undis <- dat[dat$dis == "Undiseased",]

len <- nrow(dat_undis)

dat_undis$test <- 0

dat_undis$test[1:round((1-sp)*len)] <- 1 # False +ve

dat <- rbind(dat_dis, dat_undis)

dat$test = factor(dat$test, labels=c("Negative", "Positive"))

#------------------------------------------------------------------------------

#--- Test-Negative Case-Control

d_ill <- dat[dat$ill == "Ill",] # Include only ill people

d_ <- d_ill[sample(nrow(d_ill), n),]

t <- table(d_$Vac, d_$test)

a = t[2, 2]; b = t[2, 1]

c = t[1, 2]; d = t[1, 1]

#-- VE = 1-OR

o1 <- a/b

o2 <- c/d

ve <- 1 - o1/o2

#-- Corrected VE

o1 <- (sp*(1+o1)-1)/(se*(1+o1)-o1)

o2 <- (sp*(1+o2)-1)/(se*(1+o2)-o2)

vec <- 1 - o1/o2

df <- rbind(df, data.frame(se=se, sp=sp, ve=ve, vec=vec))

}

}

ggplot(data = df, aes(x = sp, color = as.factor(se))) +

geom_hline(yintercept = 0.70, color = "gray70", linetype = "dotdash") +

geom_line(aes(y = ve), linewidth = 0.9, linetype = "dashed") +

geom_point(aes(y = ve), size = 2.5, alpha = 0.6) +

geom_line(aes(y = vec), linewidth = 0.9, linetype = "solid", alpha = 0.4) +

geom_point(aes(y = vec), size = 2.5, alpha = 0.6) +

xlab("Test Specificity") +

theme_classic() +

guides(x = "axis_truncated", y = "axis_truncated") +

scale_y_continuous(name="Vaccine Effectiveness",

limits = c(0.0, 0.8), breaks=seq(0.0, 0.8, by = 0.1)) +

theme(aspect.ratio = 6/4,

legend.position = c(0.97, 0.02),

legend.key.width = unit(1.2,"cm"),

legend.key.height = unit(0.7,"cm"),

legend.justification = c("right", "bottom"),

legend.text = element_text(size = rel(0.9)),

axis.text = element_text(size = rel(1.3), color = "black"),

axis.title = element_text(size = rel(1.6))) +

guides(color = guide_legend(title = "Test Sensitivity", order = 1))
